# Changes in the Speed–Ability Relation Through Different Treatments of Rapid Guessing

**DOI:** 10.1177/00131644221109490

**Published:** 2022-07-11

**Authors:** Tobias Deribo, Frank Goldhammer, Ulf Kroehne

**Affiliations:** 1 DIPF | Leibniz Institute for Research and Information in Education, Frankfurt am Main, Germany; 2 Centre for International Student Assessment (ZIB), Frankfurt am Main, Germany

**Keywords:** rapid guessing, response time, speed–ability relation, reliability, validity

## Abstract

As researchers in the social sciences, we are often interested in studying not directly observable constructs through assessments and questionnaires. But even in a well-designed and well-implemented study, rapid-guessing behavior may occur. Under rapid-guessing behavior, a task is skimmed shortly but not read and engaged with in-depth. Hence, a response given under rapid-guessing behavior does bias constructs and relations of interest. Bias also appears reasonable for latent speed estimates obtained under rapid-guessing behavior, as well as the identified relation between speed and ability. This bias seems especially problematic considering that the relation between speed and ability has been shown to be able to improve precision in ability estimation. For this reason, we investigate if and how responses and response times obtained under rapid-guessing behavior affect the identified speed–ability relation and the precision of ability estimates in a joint model of speed and ability. Therefore, the study presents an empirical application that highlights a specific methodological problem resulting from rapid-guessing behavior. Here, we could show that different (non-)treatments of rapid guessing can lead to different conclusions about the underlying speed–ability relation. Furthermore, different rapid-guessing treatments led to wildly different conclusions about gains in precision through joint modeling. The results show the importance of taking rapid guessing into account when the psychometric use of response times is of interest.

## Introduction

Taking the relation between test-takers’ responses and response times (RTs) into account can be helpful for multiple applications. For example, RTs can be used as collateral information for estimating latent ability (e.g., De Boeck & Jeon, 2019; Ranger & Wolgast, 2019) or enable the investigation of different cognitive processes and cognitive strategies underlying test performance (e.g., Chen et al., 2018; Scherer et al., 2015). Analogous to responses obtained under rapid-guessing behavior (R_RG_; Wise, 2017), which can bias ability estimates, it seems likely that RTs obtained under rapid-guessing behavior (RT_RG_) also introduce construct-irrelevant variance (Messick, 1995) into the measurement of test-takers latent speed. This construct-irrelevant variance may also bias the identified relationship between ability and speed. The interplay of these biases possibly leads to compromised validity of inferences based on test scores and an incorrect evaluation of the precision of estimates. Both aspects present a severe threat to inferences drawn from this data. For this reason, we examine how R_RG_ and RT_RG_ possibly affect the speed–ability relation. This study focuses on ability tests based on multiple-choice items with responses coded as correct, incorrect, or missing administered in a low-stakes setting. The present study and the results discussed are relevant to practitioners and measurement theorists concerned with response time data.

## Theoretical Background

### Rapid-Guessing Behavior

Underlying this study is the assumption that responses and their response times in a low-stakes assessment setting can be classified to be given under two different processes. These processes encompass rapid-guessing and solution behavior (Schnipke & Scrams, 1997). Test-takers may skim a task shortly but do not read and engage with a task in depth when showing rapid-guessing behavior. If a task has not been read and comprehended, a given response seems uninformative, as the response does not reflect test-takers’ ability on a specific construct. Hence, the probability of a correct response is assumed to be unrelated to a latent ability under rapid-guessing behavior. Under solution behavior, a test-taker works on a task diligently; therefore, the probability of a correct response depends on a test-taker’s ability. The impact of being in solution behavior or rapid-guessing behavior may go beyond item responses and further extend to response time data. Here, it could be assumed that while response times obtained under solution behavior RT_SB_ represent a valid measure of test-taker speed, RT_RG_ are unrelated to test-taker speed and based on another data-generating process. One data-generating process leading to RT_SB_ may encompass retrieving an answer from memory and entering the response. Another data-generating process leading to RT_RG_ may only encompass entering a response at random. For this reason, R_RG_ and RT_RG_ may introduce construct-irrelevant variance and bias test scores (Messick, 1995), as well as speed estimates and their identified relation. The following section will discuss the possible impacts of untreated R_RG_ and RT_RG_ on constructs of interest.

### The Impact of Untreated Rapid Guessing and Rapid-Guessing Response Time

**Figure 1.**
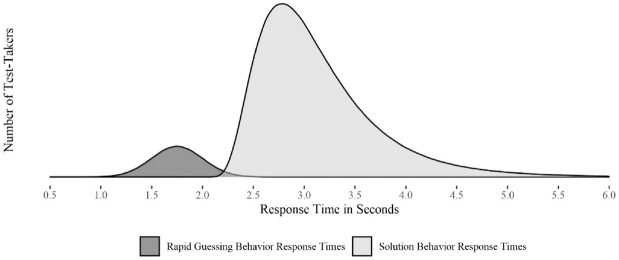
Exemplary Response Time Distributions Under Rapid Guessing and Solution Behavior.

To understand how R_RG_ does affect ability estimates, it seems helpful to keep two aspects in mind. These aspects encompass the interplay of how likely a particular response option is chosen by a test-taker when guessing and if the test-taker would have been able to solve the item if they worked on it diligently. Guttmann already noted in 1977 that the probability of choosing a specific response option while guessing is likely not equal for all response options. Recent research (Wise & Kuhfeld, 2020) corroborates this for rapidly guessed responses. Following Wise and Kuhfeld (2020), the chosen response option seems to be more strongly affected by response position than item correctness. Therefore, depending on how likely a correct or incorrect response option is chosen when guessing and how likely it is that a test-taker would have solved a task when working on it diligently, the test-taker may be more or less likely to solve it when rapidly guessing compared to when working diligently. This can lead to overestimating ability if lower ability test-takers can use guessing to solve tasks that they otherwise would not have been able to solve. Vice versa, it could lead to an underestimation of ability if test-takers appear to be unable to solve tasks they could otherwise solve if they made an effort. Moreover, producing a response under solution behavior should require more time than rapid-guessing behavior. This time difference seems reasonable, as it should take longer to read and process a task diligently than rapidly selecting an answer. Under this assumption, we expect a mixture of response time distributions related to rapid-guessing and solution behavior which possibly appear bimodal. To illustrate this and the impact of RT_RG_, see Figure 1.

Figure 1 exemplifies (based on simulated response time data) how the response time distributions supposedly differ under rapid-guessing or solution behavior. We can further try to deduce how an unidentified mixture of RTs for both processes may bias the identified RT distribution. In the presented distribution of RT_RG_, the earliest responses start around the 1-second mark. In contrast, in the distribution of RT_SB_, the earliest response starts shortly after the 2-second mark. This distribution indicates that test-takers need at least 2 seconds to comprehend and produce a solution behavior response R_SB_. Depending on the earliest possible onset of RT_SB_ and the right tail of RT_RG_, more or less overlap between both response time distributions will appear. The size of this overlap may enable a clear differentiation or make both distributions indistinguishable based on response time alone. Therefore, if both response time distributions can be distinguished, we may differentiate rapid guessing and solution behavior. For example, solution behavior starts at 1 minute and rapid-guessing behavior starts at the 1-second mark and ends at the 3-second mark. If both response time distributions start simultaneously, we cannot differentiate both processes through response time alone. Taking the distribution of response times into account, it seems likely that, depending on the onset of RT_RG_ and RT_SB_, the introduction of RT_RG_ to the overall response time distribution leads to a lower mean and an increase in variance by either extending the left tail of the overall response time distribution or making it appear heavier. The higher amount of rapid response times may also lead to an overestimation of test-taker latent speed estimates, as test-takers appear faster than they actually would have been if they had worked on an item diligently. Furthermore, prior research (Lindner et al., 2019) indicates that test-takers appear more likely to exhibit rapid-guessing behavior as the assessment progresses. Still, the onset time for the exhibition of rapid-guessing behavior varies between individuals. This variation leads to the notion that we can expect test-takers to give both responses under solution behavior and rapid-guessing behavior.

Finally, aside from the impact on individual test-takers’ effective speed and ability, we have to keep the interplay between both in mind. For example, we may assume a positive, linear relation between speed and ability in which more able test-takers solve items faster. Here, rapid guessing possibly affects the relationship in two ways. First, it may lead us to assume, for instance, a less strongly pronounced positive, linear relation compared to an unbiased relation, as the number of fast and incorrect responses increases. Second, rapid-guessing behavior may also influence the decision of which functional relation between speed and ability is assumed to be correct by influencing which model parameterization fits the observed data best. Staying with the example above, we can compare a pair of nested models. One of the models contains a positive, linear speed–ability relation and a quadratic term fixed at zero. The other model contains a curvilinear speed–ability relation by allowing the quadratic term to be estimated freely. Here, an increase in fast and incorrect responses may affect the information criteria used for model comparison and push us to choose the curvilinear model instead. Therefore, rapid guessing can lead to a mismatch between the identified and actual speed–ability relation. Both cases appear problematic as they may bias the validity of inferences based on estimates obtained by joint modeling.

### The Speed–Ability Relation in the B-GLIRT Framework

**Figure 2.**
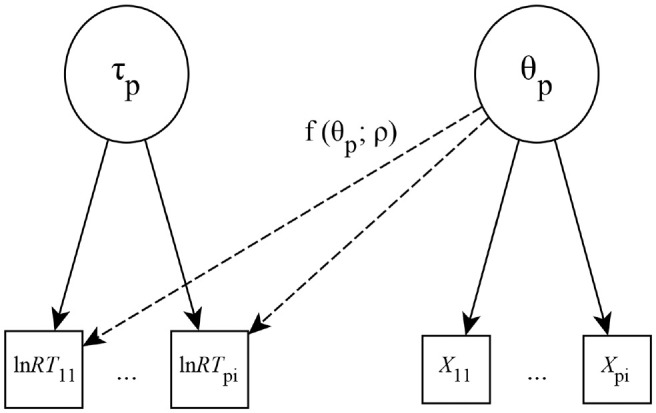
Reduced Schematic of the Bivariate Generalized Linear Item Response Theory Framework.

In recent years, psychometric models started to incorporate response times into *Item Response Theory* (IRT) models (e.g., van der Linden & Fox, 2016). Depending on the specification of the psychometric model, there are different assumptions about the functional form underlying the speed–ability relation. Most commonly, a linear (e.g., De Boeck & Partchev, 2012; Thissen, 1983; van der Linden, 2007) or a non-linear (e.g., Ferrando & Lorenzo-Seva, 2007) relation is assumed. In this article, we choose the *Bivariate Generalized Linear Item Response Theory* (B-GLIRT; Molenaar et al., 2015) framework to model the speed–ability relation, as it allows the specification of multiple, possible cross-link functions. Depending on the specification of these cross-link functions, the obtained psychometric models are equivalent (or at least sufficiently similar) to established psychometric models, which can be applied to relate response accuracy and response times. Through different specifications of the cross-link function, the B-GLIRT framework enables model comparisons of various relationships between responses and response times. In Figure 2, we find a schematic of the framework, which was reduced to only encompass directly relevant parts. For an in-depth overview and definition of the framework, we refer to Molenaar et al. (2015). The B-GLIRT framework assumes a latent ability $\theta_{p}$ and a latent speed $\tau_{p}$ for every test-taker *p*. The latent variables $\theta_{p}$ and $\tau_{p}$ are measured by responses $X_{\mathrm{pi}}$ and the log-transformed response times $\mathrm{lnR}T_{\mathrm{pi}}$ given by every test-taker *p* on every item *i*. Furthermore, at the core of the B-GLIRT framework appears the cross-link function $\;f(\theta_{p};\rho )$ . The cross-link function models the relation of test-taker latent ability $\theta_{p}$ and the cross-relation parameter vector ρ . It has to be noted that the cross-relation is only part of the response time model because the model mainly aims at increasing the measurement precision of latent ability. This also allows the framework to be equal to or similar to other popular models (e.g., van der Linden, 2007) through model parameterization. Still, it appears noteworthy that the B-GLIRT framework assumes only one underlying data-generating process for all item responses, contrary to recent mixture modeling approaches (e.g., Ulitzsch et al., 2020).

## Research Questions

Keeping the possible impact of R_RG_ and RT_RG_ on effective speed, effective ability, and the speed–ability relationship in mind, it seems possible that different (non-)treatments of R_RG_ and RT_RG_ could affect multiple points of interest. For this reason, we studied how rapid-guessing behavior affects the identification of the speed–ability relations’ functional form and the precision of ability estimates obtained. In a first step, we considered if the appearance of R_RG_ and RT_RG_ affects the functional form of the speed–ability relation compared to a reference group where we assumed less rapid guessing. In a second step, we investigated how different treatments of R_RG_ and RT_RG_ affect the functional form and the related speed and ability estimates. As a third step, we studied how different treatments of R_RG_ and RT_RG_ affect our judgment related to the increase in precision of ability estimates through joint modeling of speed and ability. Finally, in a fourth step, we investigated whether there is evidence of an increase in the validity of inferences based on test scores obtained from different treatments of R_RG_ and RT_RG_. Specifically, we evaluated whether differences appeared in predicting students’ average grades compared to the previously mentioned reference group. Here, we assumed that higher levels of *Information and Communication Technologies* (ICT) literacy should be connected to a better grade point average for the sample of students. We made this assumption based on the fact that the applied ICT literacy assessment underlies an ICT literacy proficiency model which focuses on basic ICT skills (e.g., basic software knowledge, finding and organizing information, writing e-mails, preparing informative presentations; Senkbeil et al., 2013) which appear helpful no matter the subject of study. Prior empirical research (e.g., Lei et al., 2021) also supports this notion.

## Methods

### Sample

The investigated assessment was conducted as part of the longitudinal German *National Educational Panel Study* (NEPS; Blossfeld et al., 2011). The NEPS is a panel study collecting data on competencies, educational processes, educational decisions, and returns to education in the Federal Republic of Germany following different age cohorts. The collected data are then released to the national and international scientific community to provide a database for analyses concerned with educational processes, educational research, reporting, and expert advice for policymakers in Germany. The target population of the investigated cohort was students enrolled in a public or state-approved institution of higher education in Germany. Students were followed from their enrollment in the year 2010/2011 to the year 2018. Informed consent was obtained in the recruitment process of the panel study. Here, two different modes of contact were employed. These modes were conventional mail via higher education institutions administration and personal information in lectures for freshmen students in the selected fields of studies via interviewers (Forschungsdatenzentrum des Leibniz-Instituts für Bildungsverläufe, 2021). Here, we focused on two subsamples of test-takers who participated in a computer-based assessment in 2013. The first subsample contained an unproctored individual online setting (*N_unproctored_* = 4,906). The second subsample contained a proctored group setting (*N_proctored_* = 624). Inside the unproctored setting, test-takers could work on the assessment individually without supervision (e.g., from their computer at home). In the proctored group setting, test-takers took part in the assessment with other participants in a group setting while a test-proctor managed the assessment. The assignment of the subsamples to the settings was based on a split-half design with a random selection of entire universities at which the target population had begun their studies in the winter semester of 2010/2011 (*n* = 21). These universities have been randomly administered to different settings (proctored-group vs. individual-online). Furthermore, positions of specific tests inside the assessment varied between administered rotations. Following Lindner et al. (2019), rapid-guessing behavior increases across testing time. For this reason, we decided to further differentiate the sample by assessment position (earlier or later administration) and study them separately for each setting. The two presented setting subsamples therefore were further split into a 2×2 factorial design (*N_proctored_earlier_* = 318, *N_proctored_later_* = 306, *N_unproctored_earlier_* = 2,532, and *N_unproctored_later_* = 2,374). While bias due to participation rates may appear possible (e.g., because certain subgroups are more likely to opt-out of either the proctored or unproctored setting), prior research could show that it is unlikely that the unproctored setting results in a substantially biased sample compared to the proctored setting for this specific study (Zinn et al., 2021). For this reason, it appears reasonable to assume that both subgroups resemble each other in composition and that differences in rapid-guessing behavior are induced by setting and position effects instead of outside criteria. Therefore, setting and position could be understood as mediating factors leading to differences in rapid-guessing behavior. As prior research (Kroehne et al., 2020) implies less R_RG_ inside the proctored setting (e.g., possibly due to test-takers feeling more strongly observed and scrutinized), we choose it as a reference group. For further in-depth information on the sample and the sampling process, see Zinn et al. (2017).

### Instruments/Measures

Analyses were based on data from a test of ICT literacy (Senkbeil et al., 2013) conducted as part of the NEPS. The ICT test consisted of 25 multiple-choice (MC) and five complex-multiple-choice (CMC) items. Test-takers had a fixed time limit of 29 minutes to work on the ICT assessment. No information on time progress was given in the assessment. One item was presented per screen. CMC items have been given up to two credits depending on the number of solved parts. As part of the analysis, partial credit scores on the CMC items have been dichotomized. Only a full score has been counted as correct to allow easier identification of item response times and rapid guessing thresholds.

Furthermore, we used the self-reported grade point average measured half a year after the competence assessment as a validity criterion. Grade point average was measured through a single item in which the average grade of academic achievement for the point in time had to be reported. Here, a lower value represents a better grade.

### Identification of Response Times for ICT Literacy

To obtain response times for the ICT literacy assessment, we applied a finite state machine (FSM) approach to reconstruct the test-taking process on the basis of log data (Kroehne & Goldhammer, 2018). The statistical software R (Version 4.0.5; R Core Team, 2021) and the *LogFSM* package (Kroehne, 2020) were used here. For the MC and CMC items, we differentiated two different states:

1. ICT_AS_i: Answering item *i*.
2. ICT_PS_i: Post Answering item i and navigating to *i*+1.

ICT_AS_i was identified as the period before an answer on the item *i* was given for the last time after entering the item *i*. ICT_PS_i was identified as the period after the last given answer on item *i* and leaving item *i*.

### Data Cleaning

Before conducting our analysis, we tried to filter cases that may adversely bias the underlying response time distributions. Criteria for filtering encompass missing values on all assessment items, dropping out of the assessment and prolonged inactivity. Dropping out has been defined as abortion of the assessment while participating in the ICT assessment. This abortion is indicated by no further interaction with the assessment system (and therefore no log data) after the assessment part of the ICT test (even though log data should be produced if the assessment is terminated regularly). This definition allowed differentiation between dropout and not-reaching items due to time limits. Furthermore, we filtered test-takers who did not show any interaction (e.g., clicking a button, navigating between tasks) with the assessment system for longer than 313 seconds due to inactivity. This threshold has been chosen as it is above the 99.9th percentile of test-takers item response times from all tasks of the ICT assessment. We note that multiple interactions with the assessment system normally underlie the obtained item response times, indicating that the threshold should only identify clear outliers. Overall, this reduced our sample to *N_proctored_earlier_* = 318, *N_proctored_later_* = 305, *N_unproctored_earlier_* = 2,310, and *N_unproctored_later_* = 2,176 test-takers. Interestingly, all applied filtering criteria (non-response, dropout, and inactivity) mainly identified test-takers who took part in the unproctored setting.

### Identification of Response Time Thresholds

There are multiple approaches to identify and deal with rapid-guessing behavior. Rapid-guessing behavior identification is most commonly based on a response time threshold *T_i_* or mixture modeling. We refer to Wise (2019) or Ulitzsch et al. (2020) for a more dedicated overview of rapid-guessing identification methods. This study used a response time-based threshold using the *Normative Threshold* (NT) method presented by Wise and Ma (2012). In the NT method, the threshold is set at a certain percentage (most commonly 10%) of the mean response time test-takers have taken to answer an item *i* while keeping a maximum threshold response time threshold value of 10 seconds. Here, we also used 10% of the mean response time of an item (NT10%). We have chosen the NT10% method as it presents a more conservative approach for identifying rapid guessing compared to other methods (Wise, 2017). The NT10% method is more likely to avoid misclassifying responses and response times given under solution behavior as rapid guesses. Following Wise (2017), this presents a sensible choice. While it may only allow us to identify a part of the disengaged item responses and response times, it should still improve the validity of inferences based on test scores. To identify the response time threshold *T_i_*, the total time of ICT_AS on a particular item *i* was used. The same approach was used for CMC items after the last partial response. As the user interface is identical between the proctored and unproctored settings, we also assume there is no reason to expect different response time thresholds. We compared the mean absolute difference of thresholds obtained independently in both settings to ensure this assumption.

### Treatment of Rapid Guessing

Concerning the treatment of R_RG_, different approaches prove to be of interest. These approaches appear dependent on the definition of the construct of interest. For example, in a power test, it may seem more appropriate to treat R_RG_ as incorrect (e.g., Wright, 2016) if it is assumed that all responses underlying the rapid guesses would have been incorrect. On the contrary, treating R_RG_ as not-administered in low-stakes assessments may be essential to obtain a more likely unbiased, individual measure. This is especially the case when it is unclear if a rapidly guessed item could have been answered correctly or incorrectly. Both approaches for treating rapid guessing (incorrect and not-administered) represent the possible extremes of missing value treatment (Rost, 2004). They, therefore, allow displaying the range of the impact on the identified functional form of the speed–ability relation. Other possible treatment methods have to fall in between both of these endpoints. For many applications, only test-taker speed based on a solution behavior response process (e.g., processing speed when comprehending and solving a task) seems important. The speed underlying RT_RG_ appears to be unrelated to the speed underlying RT_SB._ This means that just being able to navigate faster to the next task does not necessarily relate to being able to solve a task faster. In this case, treating identified RT_RG_ as ignorable missing values seems to be a first adequate step to eliminate the bias introduced by them into test-takers speed.

### Comparing the Speed–Ability Relation Under Different R_RG_ and RT_RG_ Treatment

We modeled the relation between latent speed and latent ability in the form of a cross-relation as presented in the B-GLIRT framework by Molenaar et al. (2015). This allowed a comparison of the cross-relations between models equivalent or sufficiently similar to the models of Thissen (1983; Regression Model), Van der Linden (2007; Hierarchical Model), De Boeck and Partchev (2012; Interaction Model), and Ferrando and Lorenzo-Seva (2007; Curvilinear Model). In the B-GLIRT framework, the model based on Thissen (1983) allows a linear relationship between speed and ability and can be extended to a model based on Van der Linden (2007) by allowing item-wise time discrimination parameters. Furthermore, the model based on Van der Linden (2007) can be extended to a model based on Ferrando and Lorenzo-Seva (2007) by allowing a quadratic cross-relation parameter or a between-subject version of the model of De Boeck and Partchev (2012) by allowing an interaction between speed and ability parameters. We applied different treatments to the identified R_RG_ and RT_RG_ in the B-GLIRT framework. The treatments encompassed treating R_RG_ as incorrect (R_RG_ = 0) or to be ignorable missing (R_RG_ = NA), as both treatments represent the possible extremes of missing value treatment (Rost, 2004). RT_RG_ is always treated to be ignorable missing (RT_RG_ = NA). Model fit is judged by *Akaike Information Criterion* (AIC) and *Bayesian Information Criterion* (BIC) for models with different functional forms. If both criteria diverge, AIC will be preferred, as the true model is not necessarily a part of the set of candidates (Vrieze, 2012). To estimate the presented B-GLIRT models, the statistical software *Mplus* (Version 8.7; Muthén & Muthén, 1998–2017) was used.

### Measuring the Precision of Estimates in MIRT Models

To compare the precision of estimates, we used the *Approximate Relative Efficiency* (ARE; de la Torre & Patz, 2005). ARE can be obtained by comparing the average posterior variance of unidimensional IRT ability estimates with the posterior variance of multidimensional IRT ability estimates, which in this case also take speed into account.

### Prediction of Criteria

We used linear regression to predict future grade point averages by the ability estimates obtained as presented beforehand for the proctored and the unproctored groups. As the self-reported grade variable had been missing for 2,964 of 5,109 (58.02 %) test-takers, we applied *Multiple Imputation* (Rubin, 1987) to deal with the missing values. For the imputation model, we used self-reported grades at two prior and two future measurement points, *Weighted Likelihood Estimates* (WLEs; Warm, 1989) of prior math and reading ability, sum scores of the five factors of personality (Rammstedt & John, 2007), and academic self-concept (Dickhäuser et al., 2002), as well as a binary gender variable. All variables were already available in the preprocessed NEPS data. We applied the *Plausible Mean Matching* algorithm for all variables. Prior and predicted grade variables had been mean-centered before conducting the imputation model. Through this, we obtained 1,000 imputed datasets that have been pooled following Rubin’s Rules (Rubin, 1987). The imputation itself has been conducted with the mice-package (van Buuren & Groothuis-Oudshoorn, 2011).

## Results

### Rapid Guesses Identification

**Figure 3.**
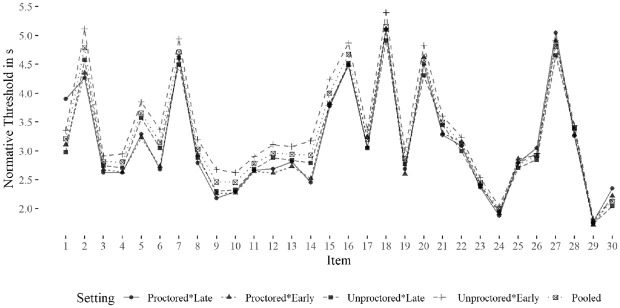
Distribution of Normative Thresholds.

We independently studied the identified thresholds for each setting and position in the first step. Overall, the thresholds appeared somewhat similar, showing a mean absolute difference of 0.09 to 0.33 seconds. Interestingly, the thresholds for the earlier position in the unproctored setting appeared later than the other combinations. The direction indicated slightly longer mean response times for the earlier position in the unproctored setting. As the thresholds of both settings appeared reasonably similar, we decided to use a pooled threshold to identify rapid-guessing behavior. For an overview regarding the identified thresholds under NT10%, see Table 1 and Figure 3.

**Table 1.** Threshold-Overview for Identified Rapid Guesses With NT10%.

Setting / Position	*N*	Threshold statistics			
		Median	*SD*	Minimum	Maximum
Proctored	623	2.85	0.91	1.75	5.10
Earlier	318	2.89	0.90	1.72	5.09
Later	305	2.80	0.91	1.77	5.11
Unproctored	4,486	3.02	0.90	1.78	5.16
Earlier	2,310	3.19	0.95	1.82	5.40
Later	2,176	2.88	0.86	1.74	4.91
Pooled	5,109	2.99	0.90	1.78	5.15

Furthermore, to obtain an overview of the distribution of individual rapid-guessing behavior, we used the *Response Time Effort* index (RTE; Wise & Kong, 2005). RTE is a measure of the individual proportion of items that have been solved under solution behavior with boundaries of zero and one. RTE = 1 indicates no rapid guessing on any item, while RTE = 0 indicates that all items have been rapidly guessed. Considering RTE values, test-takers in the unproctored setting appeared to show rapid-guessing behavior more often. Furthermore, rapid-guessing behavior seemed slightly more common if the test had been administered at a later point in time. For an overview of the distribution, see Table 2.

**Table 2.** Distribution of Response Time Effort by Setting and Position.

Response Time Effort	0	>0 and <.25	≥.25 and <.5	≥.5 and <.75	≥.75 and <1	1
	*n* (%)	*n* (%)	*n* (%)	*n* (%)	*n* (%)	*n* (%)
Proctored	0 (0)	0 (0)	0 (0)	0 (0)	4 (0.64)	619 (99.36)
Earlier	0 (0)	0 (0)	0 (0)	0 (0)	3 (0.94)	315 (99.06)
Later	0 (0)	0 (0)	0 (0)	0 (0)	1 (0.33)	304 (99.67)
Unproctored	0 (0)	10 (0.22)	23 (0.51)	40 (0.89)	225 (5.02)	4,188 (93.36)
Earlier	0 (0)	2 (0.09)	6 (0.26)	13 (0.56)	107 (4.63)	2,182 (94.46)
Later	0 (0)	8 (0.37)	17 (0.78)	27 (1.24)	118 (5.42)	2,006 (92.19)

*Note. N*_*proctored_earlier*_ = 318. *N*_*proctored_later*_ = 305. *N*_*unproctored_earlier*_ = 2,310. *N*_*unproctored_later*_ = 2,176.

### Functional Form of the Speed–Ability Relation

Table 3 contains the information criteria for selecting the best-fitting parameterization of the cross-relation function, as well as the ρ parameters which quantify the speed–ability relation in the B-GLIRT models. For the proctored setting, the AIC and BIC implied a curvilinear cross-link function based on Ferrando and Lorenzo-Seva (2007) for the earlier position and a linear function based on Thissen (1983) for the later position. The model by Ferrando and Lorenzo-Seva (2007) has been initially suggested for items from personality assessments under the distance-difficulty hypothesis, assuming that test-takers with higher positive and negative trait values respond quicker to the task. The identified cross-link function indicated a curvilinear pattern of decreasing ability with decreasing speed up to a certain point. After this point, speed increases again. Thus, high-ability and low-ability test-takers would be expected to have higher speed estimates, whereas average-ability test-takers would be expected to have lower speed estimates. We found a positive relationship based on Thissen (1983) to fit best for the latter position. Here, the linear relation in the applied model parameterization implied that high-ability test-takers worked slower than low-ability test-takers. On the contrary, the identified relation did not hold for the unproctored setting. In the unproctored setting, we identified an inverse, curvilinear relation for the earlier position at baseline. The identified functional form did match the expected functional form. The curvilinear relation still appeared more strongly pronounced than the relation in the proctored setting, as can be seen in the difference of the ρ parameters. After treatment of rapid guessing, we either found a positive, linear relation to fit best when treating R_RG_ and RT_RG_ = NA or a curvilinear relation when treating R_RG_ = 0 and RT_RG_ = NA. The identified curvilinear relation when treating rapid guessing as incorrect did appear to mirror the expected strength from the proctored setting more closely than the curvilinear relation at baseline. At the latter position, we identified an inverse, curvilinear relation at baseline. This form did not match the expected form, as the proctored setting suggested a positive, linear relation based on Thissen (1983). We appeared unable to identify the expected relation even when taking rapid guessing into account. After treating R_RG_ and RT_RG_ as not-administered, the model fit implied a model with a weaker curvilinear relation than baseline. When treating R_RG_ = 0 and RT_RG_ = NA, a model with an interaction term showed the best model fit. The model with the interaction term can be interpreted as a between-subjects version of the model presented by De Boeck and Partchev (2012). The model allows an interplay between speed and ability, which varies between test-takers. Interestingly, the positive ρ2 parameter implies more variance in the log-response times due to *θ* for slower responses. This is in line with the worst performance rule reported by Larson and Alderton (1990), which states that faster response times contain less information about test-takers’ latent ability than slower response times.

**Table 3.** Model Fit Indices for Different Cross-Relations Between Speed and Ability Under Different Rapid Guessing Treatment Approaches Split by Setting and Position.

Setting	Position	Treatment	Regression			Hierarchical			Curvilinear			Interaction			
			*AIC*	*BIC*	ρ	*AIC*	*BIC*	ρ	*AIC*	*BIC*	ρ	*AIC*	*BIC*	ρ1	ρ2
Proc.	Earlier	R_RG_ = R_RG_ / RT_RG_ = RT_RG_	19,028	19,596	1.966 (1.459)	19,526	20,094	0.248 (0.111)	**18,821**	**19,389**	**−0.065 (0.013)**	19,071	19,643	0.181 (0.007)	**−**0.006 (0.008)
Proc.	Later	R_RG_ = R_RG_ / RT_RG_ = RT_RG_	**19,733**	**20,294**	**0.795 (0.411)**	20,097	20,659	**−**0.004 (0.103)	19,871	20,432	**−**0.057 (0.020)	19,754	20,320	0.163 (0.008)	**−**0.006 (0.009)
Unproc.	Earlier	R_RG_ = R_RG_ / RT_RG_ = RT_RG_	161,216	162,084	0.450 (0.070)	161,884	162,751	**−**0.229 (0.039)	**161,189**	**162,057**	**−0.195 (0.024)**	161,732	162,606	0.187 (0.031)	0.077 (0.046)
Unproc.	Earlier	R_RG_ = NA / RT_RG_ = NA	**155,275**	**156,143**	**0.343 (0.034)**	155,782	156,650	**−**0.145 (0.038)	155,420	156,288	**−**0.060 (0.007)	155,682	156,555	**−**0.068 (0.018)	**−**0.051 (0.009)
Unproc.	Earlier	R_RG_ = 0 / RT_RG_ = NA	156,000	156,867	0.274 (0.029)	156,280	157,148	**−**0.233 (0.042)	**155,981**	**156,848**	**−0.053 (0.007)**	156,193	157,066	**−**0.058 (0.018)	**−**0.055 (0.007)
Unproc.	Later	R_RG_ = R_RG_ / RT_RG_ = RT_RG_	153,826	154,684	0.400 (0.093)	154,006	154,865	**−**0.400 (0.035)	**153,357**	**154,215**	**−0.144 (0.017)**	154,074	154,939	0.286 (0.008)	0.117 (0.006)
Unproc.	Later	R_RG_ = NA / RT_RG_ = NA	144,806	145,665	0.295 (0.037)	145,062	145,920	**−**0.294 (0.038)	**144,650**	**145,508**	**−0.114 (0.012)**	144,671	145,535	**−**0.028 (0.022)	0.314 (0.012)
Unproc.	Later	R_RG_ = 0 / RT_RG_ = NA	145,988	146,847	0.157 (0.030)	145,789	146,647	**−**0.432 (0.038)	145,660	146,518	**−**0.080 (0.008)	**145,534**	**146,398**	**−0.032 (0.014)**	**0.328 (0.022)**

*Note. N_proctored_earlier_* = 318. *N_proctored_later_* = 305. *N_unproctored_earlier_* = 2,310. *N_unproctored_later_* = 2,176. Lowest AIC/BIC values in bold. Standard errors in parenthesis. AIC = Akaike information criterion; BIC = Bayesian information criterion.

### Precision of Estimates (ARE)

The posterior variances for the unidimensional and multidimensional ability estimates, and the *ARE* of the multidimensional *B-GLIRT* models in the unproctored setting, are given in Table 4. Overall, we found that ARE indicated an increase in precision of 37% for the earlier and 43% for the later position based on the best-fitting model in the unproctored setting under the baseline condition. On the contrary, when treating identified R_RG_ and RT_RG_ as not-administered, the best-fitting model implied a slighter increase in precision of around 18% to 28% when jointly modeling responses and response times. When treating identified R_RG_ as incorrect and RT_RG_ as not-administered, the best-fitting model implied an increase in precision of 11% to 17% compared to the unidimensional estimates. The 11% to 43% increase translates to the need for around three to 13 additional items for the unidimensional model to achieve the same precision as the respective multidimensional case.

**Table 4.** Posterior Variance and Approximate Relative Efficiency for the Unproctored ICT Assessment.

Position of assessment	Threshold	Best-fitting model	R_RG_ / RT_RG_ treatment	*PV-uni.*	*PV-multi.* (ARE)
Earlier	Baseline	Curvilinear	R_RG_ = R_RG_ / RT_RG_ = RT_RG_	.349	.273 (1.37)
Earlier	NT10%	Regression	R_RG_ = NA / RT_RG_ = NA	.355	.322 (1.18)
Earlier	NT10%	Curvilinear	R_RG_ = 0 / RT_RG_ = NA	.327	.299 (1.17)
Later	Baseline	Curvilinear	R_RG_ = R_RG_ / RT_RG_ = RT_RG_	.329	.231 (1.43)
Later	NT10%	Curvilinear	R_RG_ = NA / RT_RG_ = NA	.348	.272 (1.28)
Later	NT10%	Interaction	R_RG_ = 0 / RT_RG_ = NA	.288	.259 (1.11)

*Note. N_unproctored_earlier_* = 2,310. *N_unproctored_later_* = 2,176. ICT = Information and Communication Technologies; *PV-Uni.* = Posterior Variance of the Unidimensional Model; *PV-Multi.* = Posterior Variance of the Multidimensional Model; ARE = Approximate Relative Efficiency.

### Prediction of Criteria

After we applied the presented regression models, we found a statistically significant effect of ICT literacy on self-reported grade only in our reference condition and when treating identified rapid guesses as incorrects Table 5. Furthermore, after taking rapid guessing into account, the effect sizes appeared closer to the reference group’s effect size than leaving rapid guessing untreated in the unproctored setting.

**Table 5.** Linear Regression Results.

Setting	RG treatment	Parameter	Estimate	*SE*	*df*	*p*	2.5% CI	97.5% CI
Proctored	R_RG_ = R_RG_ / RT_RG_ = RT_RG_	Intercept	**−0.120**	**0.020**	**300.5**	**<.001**	**−0.158**	**−0.081**
		ICT Literacy	**−0.053**	**0.023**	**300.5**	.**023**	**−0.098**	**−0.007**
		*R*^2^ [95% CI]	.0169 [0.0003, 0.0571]					
Unproctored	R_RG_ = R_RG_ / RT_RG_ = RT_RG_	Intercept	0.015	0.025	1,875.8	.535	**−**0.033	0.063
		ICT Literacy	**−**0.027	0.030	1,875.8	.362	**−**0.086	0.031
		*R*^2^ [95% CI]	.0004 [0.0006, 0.0044]					
Unproctored	R_RG_ = NA / RT_RG_ = NA	Intercept	0.017	0.025	1,875.8	.489	**−**0.011	0.065
		ICT Literacy	**−**0.056	0.031	1,875.8	.067	**−**0.116	0.004
		*R*^2^ [95% CI]	.0018 [0.0001, 0.0076]					
Unproctored	R_RG_ = 0 / RT_RG_ = NA	Intercept	0.019	0.025	1,875.8	.444	**−**0.029	0.067
		ICT Literacy	**−0.065**	**0.031**	**1,875.8**	.**034**	**−0.125**	**−0.005**
		*R*^2^ [95% CI]	.0024 [0.0001, 0.0088]					

*Note. N_unproctored_* = 4,486. *N_proctored_* = 623. Statistically significant parameters below α = .05 in bold. RG = rapid-guessing; 2.5% CI = lower 95% confidence interval; 97.5% CI = upper 95% confidence interval; ICT = Information and Communication Technologies.

## Discussion

The study investigated how the identification and treatment of R_RG_ and RT_RG_ affect the speed–ability relation and the related precision of ability estimates. For this, we compared the functional form of the relationship before and after rapid-guessing treatment. We used a proctored group as a reference point, assuming less rapid guessing (Kroehne et al., 2020) and, therefore, a more likely unbiased speed–ability relation. In the studied assessment, we found that a curvilinear relation between speed and ability based on a model presented by Ferrando and Lorenzo-Seva (2007) fitted best if the assessment was administered earlier in the proctored setting. This relation implies that high- and low-ability test-takers work faster than average-ability test-takers. A possible interpretation of this relation could be that test-takers showing higher values of ICT literacy already have the knowledge they need to solve the task and can recall it quickly. Test-takers with lower ICT literacy estimates may give up quickly when they realize that they do not know how to solve the task. This type of behavior appears in line with findings related to informed disengagement (Goldhammer et al., 2017). Test-takers with an average level of ICT literacy seemed to work the slowest and spend the most time on the tasks. One reason for this could be that they see a chance to solve the task by using their existing (possibly incomplete) knowledge, careful processing, or bridge inferences. On the contrary, a model with a positive, linear relation based on Thissen (1983) fitted the proctored setting best if the assessment was administered later. This relation indicates that high-ability test-takers worked slower than low-ability test-takers. An explanation for this difference might be that, due to cognitive exhaustion, high-ability test-takers cannot recall the needed information as quickly as they could if they were still rested. However, they can still solve the task when they exert a certain amount of effort. In parallel with the earlier assessment position, test-takers with lower ICT literacy estimates may also appear to have given up more quickly than the other test-takers.

While we could recover the expected functional form for the earlier position, we could not do the same for the later position even after treatment of rapid guessing. One possible reason for the misfit between the expected and identified functional form at the later position might lie in the underlying assumptions behind the treatments. We can reasonably assume that filtered rapid guessing does not appear completely at random (Rubin, 1976). Therefore, treating it as ignorable missing possibly introduces bias into the identified speed–ability relation. Furthermore, assuming that the underlying response hidden by a rapid guess would have been incorrect even if a test-taker would have tried to solve the tasks diligently seems to be a pretty harsh assumption to make. This may imply that both treatments may appear inadequate to identify the correct relation for the later position and emphasize the necessity to study the different assumptions underlying the treatment of rapid guessing (Deribo et al., 2021). The results could show how noisy response time data may appear and illustrate the need for more robust methods to estimate the speed–ability relation (e.g., Ranger et al., 2019). The findings make clear that simply ignoring rapid-guessing behavior can, but must not necessarily, lead to different conclusions about the functional form of the speed–ability relation. Even though the identified functional form may be identical, quantitative differences may appear concerning the strength of the speed–ability relation itself. This could be seen in the change of the *ρ* parameters between the models. Identification and treatment of R_RG_ and RT_RG_ were shown to recover the expected functional form at least in part.

We found that incorporating response times and applying a joint estimation of speed and ability increased precision for all models. Different (non-)treatments of R_RG_ and RT_RG_ led to different conclusions about gains in precision through joint modeling. The increase varied from 11% to 43%, translating to the need for roughly three to 13 items to achieve the same level of precision with a unidimensional model. These wildly different findings appear important to note, especially in the context of large-scale assessment. In this context, multiple cognitive assessments and questionnaires are to be administered, and testing time and space for each part of the test are strongly limited. We note that all models with untreated rapid-guessing behavior showed the highest increase in efficiency through joint modeling of speed and ability. Therefore, rapid-guessing behavior does not necessarily have to be expected to show a detrimental effect on accuracy. It appears possible that rapid-guessing behavior can increase the size of the relation between speed and ability, which may also lead to an increase in measurement accuracy. While the models with untreated rapid-guessing behavior showed the highest increase in accuracy, this likely comes at the expense of the validity of the underlying construct and its interpretation. This expense appears evident, for example, if we define a construct of cognitive processing speed, which is measured by the time a test-taker spent on processing a stimulus and retrieving the related information out of memory. In this case, RT_RG_ does not capture the process of interest, and the obtained speed estimate does not reflect the desired construct. The problem harkens back to the notion that reliability is a necessary but not sufficient condition for validity. We emphasize that while we found differences in the precision of the models, the choice of rapid-guessing treatment appears to be the foremost one of validity. Even though a particular treatment or non-treatment may lead to higher precision, the choice should be guided by which treatment allows the most valid inferences based on test scores. In light of which rapid-guessing treatment we judge to be valid, we may have to adjust our expectations related to the increase in precision obtained through jointly modeling speed and ability accordingly. In the presented case, we also found a statistically significant effect of ICT literacy estimates on future self-reported grades only in our reference group or after identifying and treating rapid-guessing behavior as incorrect. Based on prior research (Lei et al., 2021) and our reference group, we would expect a small relation between both variables for university students. As prior research could show that the responses underlying the identified rapid guesses are more likely to be incorrect in this specific use case (Deribo et al., 2021), it appears possible that treating rapid guesses as incorrect may best approximate the responses which would have been given if the test taker did not rapidly guess. The results can present at least some evidence for higher validity for inferences based on test scores after rapid guessing has been taken into account. While the biasing nature of rapid guessing appears to be evident for obtained ability estimates, the same can be said for speed. Suppose we want to interpret speed substantively. In that case, we have to have a clear understanding of the underlying processes and the construct and possibly need to be aware of the potentially biasing nature of rapid guessing. Altogether, the results show the importance of taking rapid guessing into account when the psychometric use of response times is of interest.

## Limitations

One of the study’s main limitations is the possibility that the responses identified as rapid guesses only represent a fraction of disengaged responses. It appears possible that responses that have been false-negatively classified as obtained under solution behavior are actually disengaged. Extending this to our groups, while we can see a clear difference in rapid-guessing behavior between the proctored and unproctored groups, this does not necessarily mean that the proctored group is more engaged. In this line of thought, we note that other response strategies, styles, and types, for example, informed guessing (e.g., Goldhammer et al., 2017), appear possible. These strategies may also affect the speed–ability relation in an unintended way, and their effect on the validity of test-scores has to be critically reflected. Therefore, it appears possible that the proctored group only showed a different kind of disengagement, which is non or not as rapid. Furthermore, the study assumes that differences in rapid-guessing behavior are mediated by setting and test position. While prior research (Zinn et al., 2021) and the applied sampling process (random selection of universities) support this notion, future research should consider how rapid guessing could be experimentally induced and more tightly controlled, for example, through instruction or incentivization. Another limitation lies in the treatment of R_RG_ and RT_RG_. While treating R_RG_ as incorrect or ignorable missing allows to capture the extreme points of missing value treatment (Rost, 2004), other approaches may allow for a more valid measure of ability itself (e.g., Deribo et al., 2021; Liu et al., 2019). It remains unclear what the best approach is to deal with filtered RT_RG_. One possibility may be replacing filtered response times with more sophisticated methods like Multiple Imputation. Furthermore, four out of five CMC items of the assessment have been dichotomized, possibly leading to a certain loss of information that may affect the model estimation and fit indices. Finally, while the findings related to the best-fitting speed–ability relation may appear ambiguous, we want to reaffirm that we are dealing with a still open research field. Even though a systematic approach may appear helpful to get closer to the underlying methodological problem, we have not yet come far enough to draw precise, generic conclusions from it. This is especially true as the present study focuses only on one specific domain (ICT literacy). To obtain a better understanding of the topic and generalize the results, it appears necessary to extend this line of research to other assessment domains (e.g., math, reading, or intelligence tests), other types of assessment (e.g., questionnaires), and other assessment contexts (e.g., high-stakes or strongly time-limited assessments) especially if there is the possibility of strong prior assumptions about the supposed relation between speed and trait.
